# Genetic variability in HPV 33 and 35 E6 and E7 genes from South African and Mozambican women with different cervical cytology status

**DOI:** 10.1186/s12985-025-02851-2

**Published:** 2025-07-10

**Authors:** Cremildo Maueia, Alltalents T. Murahwa, Olivia Carulei, Ongeziwe Taku, Zizipho Mbulawa, Alice Manjate, Ziyaad Omar Valey, Tufária Mussá, Anna-Lise Williamson

**Affiliations:** 1 Division of Medical Virology, Department of Pathology, Faculty of Health Sciences, University of Cape Town, Cape Town, South Africa; 2 Departamento de Microbiologia, Faculdade de Medicina, Universidade Eduardo Mondlane, Maputo, Mozambique; 3 Instituto Nacional de Saúde, Maputo, Mozambique; 4 Centre for the AIDS Programme of Research in South Africa (CAPRISA), Nelson R Mandela School of Medicine, University of KwaZulu-Natal, Durban, South Africa; 5 Department of Laboratory Medicine and Pathology, Nelson Mandela Academic Laboratory (NHLS), Walter Sisulu University, Mthatha, South Africa; 6 National Health Laboratory Service, Nelson Mandela Academic Hospital, Mthatha, 5100 South Africa; 7 National Health Laboratory Service (NHLS), Virology and Tissue Immunology, Groote Schuur Hospital, Cape Town, South Africa; 8 Institute of Infectious Disease and Molecular Medicine, University of Cape Town, Cape Town, South Africa

**Keywords:** HPV 33/35 variability, Phylogeny, Cytology

## Abstract

**Background:**

Among the high-risk human Papillomavirus (hr-HPV) genotypes related to cervical cancer (CC) cases, HPV16 and 18 are the most studied worldwide. However, several studies have identified HPV 33 and HPV 35 as some of the most common genotypes in sub-Saharan African regions. This study aims to investigate the genetic variability and lineages of HPV 33 and 35 based on the HPV E6 and E7 genes in isolates from South African and Mozambican women with different cervical cytology statuses.

**Methods:**

The study analysed 26 HPV 33 and 46 HPV 35 DNA samples collected previously from South African and Mozambican women. The E6 and E7 genes were amplified by polymerase chain reaction (PCR) using genotype-specific primers. Sequences were mapped to the reference sequences using Qiagen CLC Genomics Workbench software and aligned with the HPV 33 and 35 lineages reference sequences. Single nucleotide polymorphisms (SNPs) in the E6 and E7 genes were identified, and the phylogenetic trees were generated.

**Results:**

Of the 26 HPV 33-positive subjects, 62% (16/26) were from women with high-grade squamous intraepithelial lesions (HSILs). Phylogenetic analysis revealed that 38% (10/26) of the isolates clustered with European lineages. Specifically, 30% (8/26) of isolates clustered in the A1 sublineage, and 8% (2/26) in the A2 sublineage. The African 1 lineage (B1 sublineage) was identified in 19% (5/26) of the isolates. Notably, 42% (11/26) of the isolates did not cluster with any of the reference sequences under investigation, through E6 and E7 genes analysis. In the HPV 33 E6 gene, 80 SNPs were identified and 30 in the E7, frequently in the HSILs subjects. Of the 46 HPV35-positive subjects, 46% (21/46) were from women with HSILs, and 43% (20/46) of the isolates clustered with the European lineages. Specifically, 39% (18/46) clustered to the A1 sublineage, and 4% (2/46) clustered to the A2 sublineage. However, 4% (2/46) of the isolates did not cluster with any of the study sublineage. Seven SNPs were detected in the E6 region and two in the E7 region of the HPV 35 isolates.

**Conclusion:**

The present study’s genetic analysis showed a higher prevalence of European HPV 33 and 35 variants. Fewer SNPs were found in the studied genes of HPV 35 isolates. The addition of HPV 35 to the HPV vaccines would result in improved cervical cancer prevention. The study findings contribute to a better understanding of the genetic diversity of the HPV circulating in Southern African women and may inform strategies for cervical cancer prevention, including the design of therapeutic vaccines for women in advanced cytological disease stages.

**Supplementary Information:**

The online version contains supplementary material available at 10.1186/s12985-025-02851-2.

## Introduction

Since the establishment of human papillomavirus (HPV) as the primary cause of cervical cancer (CC), certain HPV genotypes have been identified as associated with cervical cancer and are classified as high-risk HPV (hr-HPV) genotypes [1]. The hr-HPV group includes 15 different types: HPV 16, HPV 18, HPV 31, HPV 33, HPV 35, HPV 39, HPV 45, HPV 51, HPV 52, HPV 56, HPV 58, HPV 59, HPV 68, HPV 73, and HPV 82 [1, 2].

HPV has a circular double-stranded DNA genome of approximately 8000 base pairs, encoding 2 separate groups of viral proteins: the early genes (E1, E2, E4, E5, E6, E7, E8) and the late genes (L1, L2). The early genes are involved in viral replication, transcriptional regulation, and oncogenesis [2, 3]. The E6 and E7 genes encode viral oncogenes necessary for malignant transformation, as they inactivate the tumour suppressor proteins p53 and pRb, respectively, leading to cellular transformation [1, 3]. Each unique HPV type is defined by a minimum 10% difference in the highly conserved L1 nucleotide sequence compared to other characterised types [4, 5].

Each hr-HPV genotype can be divided into lineages and/or variants and multiple sublineages [6]. The distribution of these genetic subtypes varies significantly by geography, ethnicity, and age group, which may lead to some differences in cervical lesion type and prevalence in infected populations [6, 7]. Variations between genetic subtypes result from genetic differences that can alter amino acid sequences, which can, in turn, affect the virus’s oncogenic potential, host immune response, and effectiveness of current HPV vaccines [7, 8].

Among hr-HPV genotypes, HPV 16 and 18 are the most studied and included in all available HPV vaccines as they account for approximately 75% of CC cases worldwide [9]. However, HPV 33 and HPV 35, though less frequently detected in North America and Europe, are commonly identified in sub-Saharan Africa [10, 11]. For example, studies in Mozambique identified HPV 33 and 35 to be among the most common hrHPV types after HPV 16 and 18 in cervical samples. Similarly, studies in Malawi and South Africa found that HPV 33 and 35 were among the most frequently detected hrHPV types in cervical cancer cases, as they ranked third or fourth in frequency after HPV16 and/or HPV18 [12, 13, 14]. In rural Zimbabwe, HPV 33 and 35 were also frequently identified in cervical cancer cases [15, 16].

HrHPV oncoproteins such as E6 and E7 are potential targets for therapeutic vaccine development [17] using a combination or single genes [18]. While the current therapeutic vaccines are largely targeting cervical cancer, there is an interest in ones for all stages of HPV infection [17, 18]. Understanding variation in the hr-HPV E6 and E7 genes is essential, as this may impact the efficacy of these vaccines [18]. Nonsynonymous mutations in these genes have been widely reported in HPV infections worldwide [17, 19]. These mutations can alter protein structure and function, affecting virus viability, carcinogenic potential, and interactions with host cells [7, 19]. In addition, HPV 33 is targeted by the Gardasil nonavalent vaccine, while HPV 35 is not included in any HPV vaccines currently in use [20].

HPV 35 is one of the most prevalent genotypes in sub-Saharan Africa after the HPV 16 and 18 genotypes [21]. However, sequence data on circulating African lineages of HPV 35 remains limited. Understanding the genetic diversity and circulating variants of HPV33 and 35 in specific geographical regions is essential for the HPV vaccine design and optimisation, since it is part of cervical cancer prevention and treatment strategies [22]. South Africa and Mozambique are two countries in the Southern Africa region with a distinct population structure and diversity, as well as a high cervical cancer burden. To add to this, a higher prevalence of the Human Immunodeficiency virus (HIV) infection, which can impact the HPV infection, is notable in these two countries [21]. Despite the relatively high prevalence of HPV 33 and HPV 35 in sub-Saharan Africa, limited genomic data are available on the E6 and E7 gene variability in these populations. This study aimed to investigate the genetic variability and lineages of HPV 33 and 35 by analysing the E6 and E7 genes in South African and Mozambican women with different cervical cytology statuses.

## Materials and methods

### Population study and samples

This retrospective molecular epidemiology study used archived cervical samples collected across multiple healthcare settings, including cervical cancer screening and general medical services in South Africa and Mozambique. From a total of 500 women, samples positive for HPV 33 and HPV 35 were selected based on prior genotyping. No other HPV types were included. The inclusion and exclusion criteria can be accessed from Taku et al. (2020), Maueia et al. (2021) and Mbulawa et al. (2024). Informed consent was given to store specimens for future HPV studies. Table 1 summarises the study sites, participant groups, and sample collection procedures. Symptomatic and asymptomatic women were included. All the study samples were stored at -80 °C until the HPV genotyping. Pap smears and biopsies (where necessary) were performed to assess the cervical cytological abnormalities classified according to the 2001 Bethesda System.

**Table 1:** Summary of study population and sample collection

Location	Study Site	Timeframe	Population	Sample Collection Method	HPV Genotyping Method
South Africa (Eastern Cape, OR Tambo District, Mthatha) [14]	Community Health Clinic	09/2017–08/2018	Women seeking cervical cancer screening or general medical care.	Cervical brushes.	Multiplex HPV Direct Flow CHIP Kit (Vitro Master Diagnóstica, Sevilla, Spain)
South Africa (Eastern Cape, OR Tambo District, Mthatha) [23]	King Sabata Dalindyelo (KSD) Health Facility	06–07/2023	Women seeking medical care for various conditions or medication collection.	Cervical brushes.	Seegene Anyplex™ II HPV28 (Seegene Inc., Seoul, South Korea)
Mozambique (Maputo, Mavalane Health Area) [24]	Public Health Facilities	02/2018–07/2019	Non-pregnant women with gynecological symptoms (e.g., venereal pain, genital ulcers, vaginal discharge) or seeking family planning services.	Cervical brushes.	Multiplex HPV Direct Flow CHIP Kit (Vitro Master Diagnóstica, Sevilla, Spain)

### DNA extraction and HPV genotyping

HPV DNA extraction was performed using a MagNA Pure Compact (Roche Diagnostics, Indiana, USA) and the MagNA Pure Compact Nucleic Acid Isolation Kit (Roche Diagnostics, Indiana, USA) following the manufacturer’s instructions. Since samples were collected and genotyped in two different settings, HPV genotyping was performed using two methods, and both accurately detects HPV 33 and HPV 35 and were used under quality-controlled conditions:

1. Multiplex HPV Direct Flow CHIP Kit (Vitro Master Diagnóstica, Sevilla, Spain).
2. Seegene Anyplex™ II HPV28 (Seegene Inc., Seoul, South Korea) multiplexed real-time polymerase chain reaction (PCR).

Genotyping was based on amplifying a fragment in the HPV L1 region, followed by hybridisation using DNA-specific probes using DNA-Flow technology for manual HybriSpot platforms according to the manufacturer’s instructions. DNA samples that became positive for the HPV 33 and 35 genotypes, were selected. For the present study, the full length of the E6 and E7 genes were sequenced due to their roles in the HPV-mediated carcinogenesis, which offers improved lineage resolution and potential mutation insights as well as their higher genetic variability compared to L1.

### DNA amplification and sequencing

From the HPV 33 and 35-positive previously genotyped samples, the full-length of the E6 and E7 genes were amplified by PCR using two primer pairs specific for each genotype, designed using Geneious Prime (Dotmatics, V.2024.0.5) bioinformatics software. To minimise batch effects, all molecular and bioinformatic analyses were performed in a single laboratory setting. The primers were based on GenBank HPV reference sequences for HPV 33 (accession M12732) and HPV 35 (accession X74477). The specific primers for HPV 33 and HPV 35 sequences are listed in Table 2. The PCR amplification of each gene was first performed in a 50 µl reaction volume containing 5µL of each primer, 12,5µL of water, 25µL of Read Taq Mix buffer (Meridian Bioscience, Ohio, USA), and 2,5µL of the template sample. Thermocycling conditions used were: 95 ◦C for 10 min followed by 40 cycles of 95 ◦C for 1 min, 55 ◦C for 1 min, 72 ◦C for 1 min, and final elongation at 72 ◦C for 5 min. A positive control (composed of HPV 33 and 35 known positive samples) and a negative control (water) were included in the mix. A two per cent agarose gel stained with Ethidium bromide solution (Sigma Aldrich, Missouri, USA) was used to visualise the amplicons.

**Table 2:** Primers for human papillomavirus (HPV) 33 and 35 sequence analysis

Genotype	Primer	Sequences 5’– 3’	Amplicons Length (bp)
HPV 33	49 F	GTTCAACCGAAAACGGTGCA	780
	833R	CACTGTGCCCATAAGTAGTTGC	
HPV 35	24 F	ACCGAAAACGGTCGTACC	830
	836R	CACACTATTCCAAATGTGCCCA	

### Illumina data pré-library quality control and assembly

From a total of 119 samples (47 HPV 33 and 71 HPV 35) that were submitted for quality control (QC) analysis, through PCR product band confirmation, 72 samples passed all quality control assessments. The remaining 47 samples were excluded due to QC failure based on criteria such as: no amplification observation, multiple fragments presence and incorrect fragment size observation.

For the library preparation, an individual concentration of the libraries ranged from 1.80– _11.58 ng/µl, and the average fragment size distribution ranged from 393 to 569 base pairs (bp). The individual libraries were diluted and pooled at equimolar concentrations. The final pool had an average fragment size of 475 bp and a final concentration of 4 nanomolar (nM) that was further denatured and diluted to a final loading concentration of 8 pM on the Illumina MiSeq. The sequencing run metrics were obtained from Illumina Sequencing Analysis Viewer (SAV) after sequencing. The sequencing run was completed successfully without errors, and the percentage of bases with a quality score of 30 or higher (Q30) was above 70%, which is the recommendation made by Illumina.

### Library preparation, quality control and pooling

The libraries were prepared from 72 DNA samples (26 HPV 33 and 46 HPV 35) using the Illumina DNA Prep kit according to the manufacturer’s instructions. A DNA input of 250 ng was used for each sample. Briefly, the library preparation consisted of the following steps: Tagmentation of genomic DNA and bead cleanup, Indexing and PCR amplification of tagmented DNA. The average fragment size distribution for each library and pooled libraries was determined using the TapeStation D1000 High Sensitivity Screen Tape Assay (Agilent, Santa Clara, USA). The individual libraries were quantified using the QuantiFluor ONE dsDNA kit (Promega, New York, USA). The concentration of adapter-ligated DNA molecules was confirmed in the final sequencing pool using the NEBNext^®^ qPCR Library Quant Kit (New England Biolabs, London, UK).

Nucleotide sequences were obtained using 300 bp paired-end sequencing with the Illumina MiSeq system and MiSeq Reagent Kit v3 (600 cycles) (Illumina, San Diego, USA) according to the manufacturer’s instructions. The quality of the sequenced fragments, corresponding to the 780 bp and 820 bp regions covering the E6 and E7 ORFs of HPV 33 and 35, respectively, was analysed using FastQC software (Babraham Bioinformatics, V 0.12.0). All sequences showed a sufficient quality score (> 99.99%) after their analysis repetition. Illumina sequence reads were processed using CLC Genomics Workbench (Qiagen, Germany) due to the high size of the DNA fragments. Reads were trimmed using a quality score limit of 0.05, and any reads with more than 2 ambiguities were discarded. Reads were assembled using the assembly against reference function with default parameters and sequences trimmed to cover only the E6 and E7 regions studied nucleotides in CLC Genomics Workbench (Qiagen, Germany).

### Phylogenetic and statistical analyses

Study sequences were paired, trimmed, and mapped to the reference sequence, and then consensus sequences were extracted using Qiagen CLC Genomics Workbench software (Qiagen, V 24.0.2). The consensus sequences were aligned with 13 reference sequences belonging to HPV 33 sublineages identified in the sub-Saharan Africa studies: M12732, HQ537688, HQ537698, HQ537694, HQ537699, OP712099, OP970997, OP971069, and EU918766 from A lineage (European variants); HQ537705, HQ537706, and OP712072 from B lineage (African 2 variant) and KF436865 from C lineage (African 1 variant). For HPV 35, consensus sequences were aligned with 19 reference sequences from A lineage (European variants): 11 reference sequences from A1 sublineages (X74477, HQ537710, HQ537712, HQ537713, HQ537715, HQ537717, HQ537719, MN829882, OP712067, OP971063 and OP971100) and eight from A2 sublineages (HQ537721, HQ537723, HQ537724, HQ537725, HQ537727, HQ537728, MN829881 and OP712025). All the reference sequences were obtained from Papilloma Episteme (PaVE, https://pave.niaid.nih.gov, accessed on 24 October 2024). Nucleotide alignment was performed using Geneious Prime software (Dotmatics, V.2024.0.5) from which the primers were designed. Maximum Composite Likelihood phylogenetic trees were generated in MEGA 11 [25] using the UPGMA method [26]. The reliability of the observed clades was proved with internal node bootstrap values of ≥ 50% (after 1000 replicates). Demographic data were organised in an Excel database. Tables were generated using Microsoft Excel 2017, and graphics were generated using GraphPad Prism version 7.2 (GraphPad Software, San Diego, CA).

The HPV 33 and 35 prevalence and lineages were calculated. Categorical variables were summarised using percentages as appropriate. When data were presented as proportions of the total sample, the missing data were excluded from the denominator. The Prisma Chi-square test was used to determine the statistical significance of differences in lineage distribution between groups with positive and negative cytology results. Statistical tests were considered significant if the *p*-value was equal to or less than 0.05.

All sequences were submitted to the NCBI database, and their accession numbers in GenBank range from 246,762 to 246,787 for HPV 33 and from 246,788 to 246,833 for HPV 35.

## Results

### Study population

The study population characteristics can be accessed in Taku et al. 2020 [14] and Mbulawa et al. 2024 [23] for South African participants and in Maueia et al. 2021 [24] for the Mozambican participants. Of the 47 HPV 33-positive samples, 55% (26/47) yielded high-quality sequences (≥ 99% coverage) suitable for inclusion in downstream analysis and of 61 HPV 35-positive samples, 75% (46/61) had a score quality suitable for inclusion in downstream analysis. The median interquartile range (IQR) age of the study participants was 46 (38–55) years for the South African and 38 (14–62) for the Mozambican study population. The cytology results are given in Table 3.

**Table 3:** Cytology results of the HPV 33 and 35-positive study participants

	HSIL *n* (%)	non HSIL *n* (%)	NILM *n* (%)
HPV 33 (*N* = 26)	16 (62)	7 (27)	3 (11)
HPV 35 (*N* = 46	21 (46)	7 (15)	18 (39)

HSIL—High-grade squamous intraepithelial lesion, non-HSIL–any cytological condition other than HSIL (NILM—negative for intraepithelial lesion or malignancy)

### HPV 33 phylogenetic analyses

**Fig. 1:**
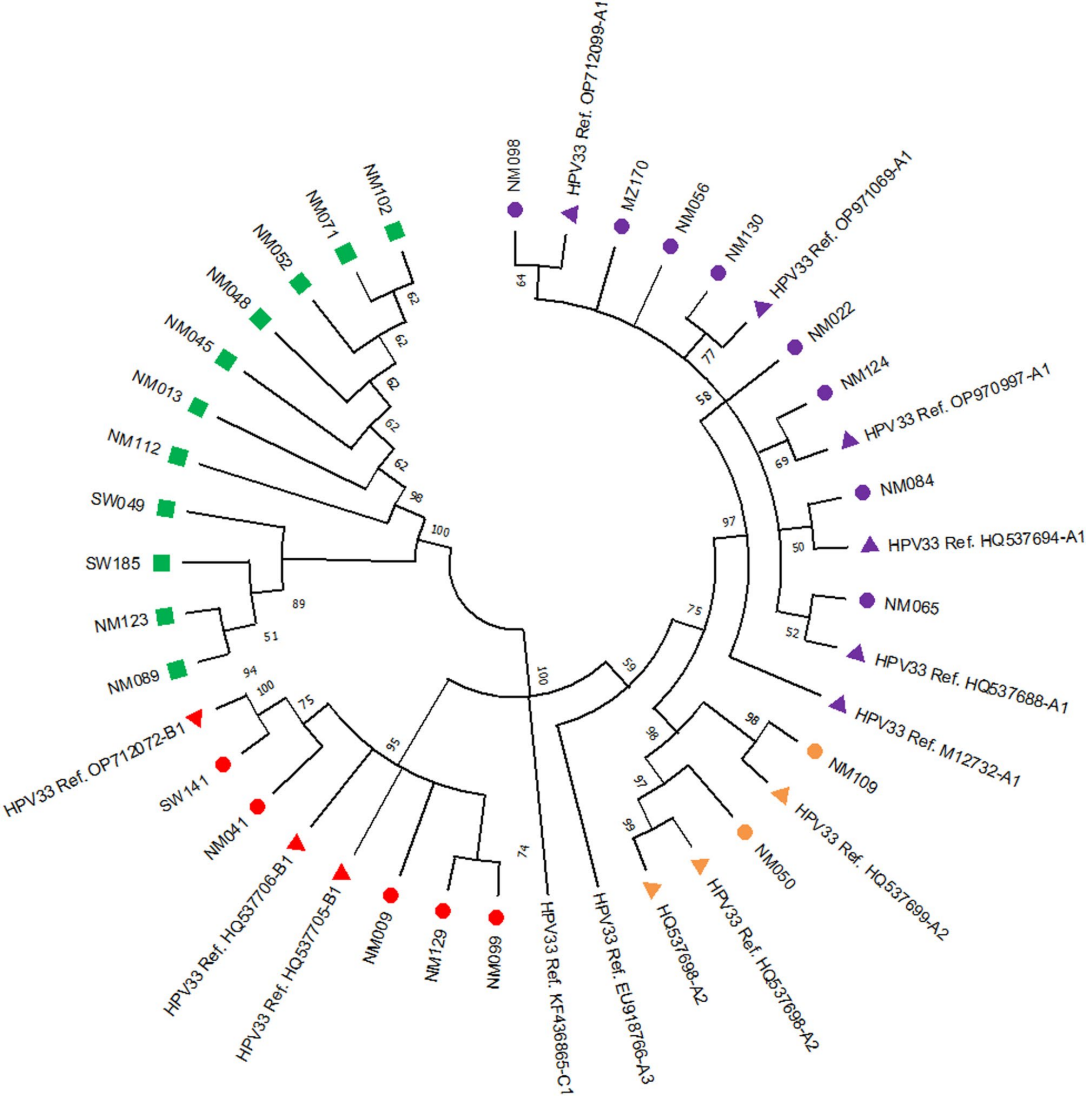
HPV 33 E6 and E7 genes tree. The phylogenetic relationship of HPV 33 sequences aligned with five reference sequences. The evolutionary history was inferred using the UPGMA method [26]. The bootstrap consensus tree was generated from 1000 replicates representing the evolutionary history of the taxa analysed [27]. Branches corresponding to partitions reproduced in fewer than 50% of bootstrap replicates were collapsed. The evolutionary distances were computed using the Maximum Composite Likelihood method [28] and are in the units of the number of base substitutions per site. The evolutionary analyses were conducted in MEGA 11 [29], and the equal-rates Markov (ERM) model was the evolutionary model applied. NM represents samples collected at Nelson Mandela Academic Hospital, SW represents the samples collected at King Sabata Dalindyelo (KSD) health facility (South Africa), and MZ represents the samples collected in Maputo (Mozambique). Sublineages and clusterings are indicated in coloured nodes: A1: purple, A2: orange, B1: red, and non-clustered: green square nodes. Reference sequences are shown in triangle nodes

Phylogenetic analysis of the 26 HPV 33 isolates aligned with reference sequences (Fig. 1) showed that 38% (10/26) of all isolates belonged to the A lineage (European variants). Specifically, 30% (8/26) of isolates clustered in the A1 sublineage, and 8% (2/26) in the A2 sublineage. Additionally, 19% (5/26) belonged to the B lineage (African 2 variant, B1 sublineage). Notably, 42% (11/26) of the isolates grouped together on one branch, did not cluster with any of the reference sequences included, and no isolates matched the remaining sublineages under investigation.

### HPV 33 lineages distribution according to the cytology

Of the 26 HPV 33 isolates, 62% (16/26) were collected from women diagnosed with HSILs as a cervical abnormality (Table 4). Of the ten subjects clustered into European variants, 23% (6/26) were collected from women with HSILs and 11% (3/26) from women with non-HSIL abnormalities. Among the isolates that did not cluster with any of the HPV 33 reference sequences, 27% (7/26) were from participants with HSILs.

**Table 4:** Distribution of the HPV 33 lineages by the cytology

HPV 33 lineages	Cytology			
	HSIL *n* (%)	No-HSIL *n* (%)	NILM *n* (%)	Total (%)
A (European)	6 (23)	3 (11)	1 (4)	10 (38)
B (African 1)	3 (11)	2 (8)	0	5 (19)
Not clustered	7 (27)	2 (8)	2 (8)	11 (42)
Total (%)	16 (62)	7 (27)	3 (11)	26 (100)

HSIL—High-grade squamous intraepithelial lesion, non-HSIL–any cytological condition other than HSIL (LSIL—low-grade squamous intraepithelial lesion and ASCUS—atypical squamous cells of undetermined significance), NILM—negative for intraepithelial lesion or malignancy

### HPV 33 E6 and E7 SNPs

The HPV 33 study sequences were compared to the five reference sequences from the HPV 33 sublineages to analyse the presence of single nucleotide polymorphism (SNPs) in the E6 and E7 regions. All study sequences contained more than one SNP. The E6 region showed a total of 79 SNPs, and the E7 region showed a total of 30 SNPs (Supplementary Material 1). Notably, 68 of the most frequently detected SNPs in the E6 region and 26 in the E7 region were found in isolates that did not cluster with any HPV 33 reference sequences (Supplementary Material 1).

Compared to the HPV 33 reference sequence (GenBank: M12732), all the HPV 33 isolates showed nucleotide variation in the E6 fragment. In the E7 fragment, 11(42%) isolates showed a complete sequence homology with the reference and the remaining 15 (58%) isolates showed nucleotide variation. All the E7 nucleotide variations were non-synonymous substitutions, while in the E6, 16 were non-synonymous mutations that resulted in some amino acid changes. No mutations generating a frameshift or a premature stop codon were observed.

### HPV 35 phylogenetic analyses

**Fig. 2:**
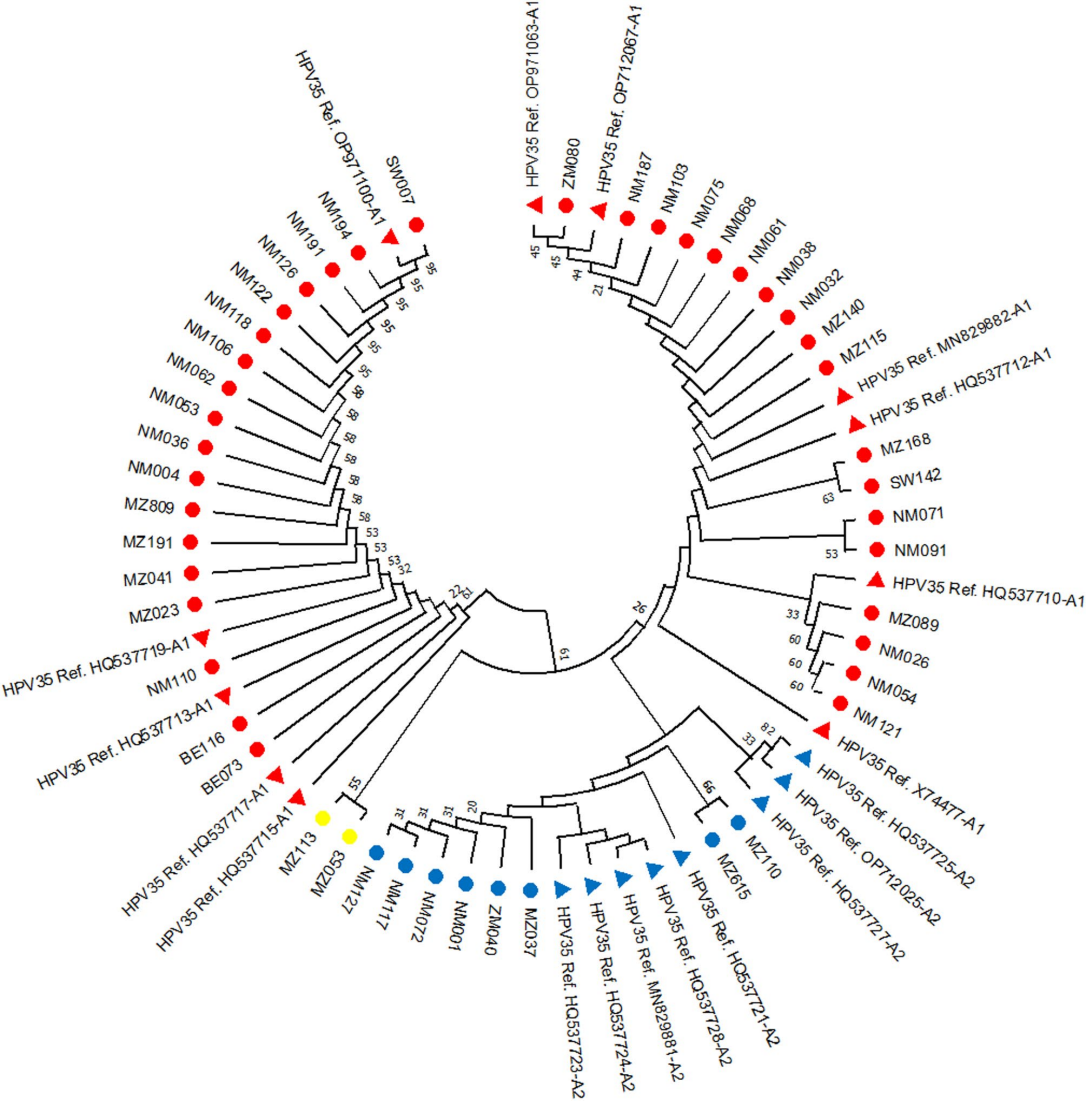
HPV 35 E6 and E7 genes tree. The phylogenetic relationship of HPV 35 sequences aligned with 19 reference sequences of HPV 35. The evolutionary history was inferred using the UPGMA method [26]. The bootstrap consensus tree inferred from 1000 replicates represents the evolutionary history of the taxa analysed [27]. Branches corresponding to partitions reproduced in less than 50% of bootstrap replicates were collapsed. The evolutionary distances were computed using the Maximum Composite Likelihood method [28] and are in the units of the number of base substitutions per site. The evolutionary analyses were conducted in MEGA 11 [29], and the equal-rates Markov (ERM) model was the evolutionary model applied. NM represents samples collected at Nelson Mandela Academic Hospital, BE represents the samples collected at Mbekueni Community Clinic, SW and ZM represent the samples collected at King Sabata Dalindyelo (KSD) health facility (South Africa), and MZ represents the samples collected in Maputo (Mozambique). Sublineages and clusterings are indicated in coloured nodes: A1: red, A2: blue, and non-clustered: yellow nodes. Reference sequences are shown in triangle nodes

Of the 46 HPV 35 subjects aligned to the European variants reference sequences, 78% (36/46) were clustered to the A1 sublineages, and 16% (8/46) were clustered to the A2 sublineage (Fig. 2). This study found that 4% (2/46) of the isolates did not match any of the HPV 35 reference sequences under investigation.

### HPV 35 lineages distribution according to the cytology

Table 5 shows that of the 46 study participants with HPV 35, 46% (21/46) had HSILs as a cervical abnormality, and 39% (18/46) had no abnormality. Of the 21 participants with HSILs, 39% (18/46) had an HPV 35 clustering to the A1 sublineage, 4% (2/46) clustering to the A2 sublineage and 2% to an unknown sublineage.

**Table 5:** Distribution of the HPV 35 lineages by cytology

HPV 35 lineages	Cytology			
	HSIL *n* (%)	No-HSIL *n* (%)	NILM *n* (%)	Total (%)
European A1	18 (39)	5 (11)	13 (28)	36 (78)
European A2	2 (4)	2 (4)	4 (8)	8 (16)
Non-clustered	1(2)	0	1(2)	2(4)
Total (%)	21 (46)	7 (15)	18 (39)	46 (100)

HSIL—High-grade squamous intraepithelial lesion, non-HSIL–any cytological condition other than HSIL (LSIL—low-grade squamous intraepithelial lesion and ASCUS—atypical squamous cells of undetermined significance), NILM—negative for intraepithelial lesion or malignancy

### HPV 35 E6 and E7 regions nucleotide sequences SNPs

The HPV 35 sequences were compared to the two reference sequences from the HPV35 sublineages to analyse the presence of single-nucleotide polymorphisms (SNPs) in the E6 and E7 regions. Seven SNPs were detected in the E6 region and two in the E7 region (Table 6), and all sequences showed the presence of more than one SNP. The most frequently detected SNPs in E6 and E7 are shown in Table 6. All the mutations were synonymous, and did not result in any amino acid changes, no mutations generating a frameshift, or a premature stop codon were observed in both E6 and E7 fragments.

**Table 6:** Nucleotide sequence variations in the E6 and E7 of the HPV35 samples (*n* = 46)

Region	Nucleotide position	Nucleotide change	*n*	Functional annotation	Amino acid change	%	Clustering sublineage
E6	**127**	**T > C**	**18**	Synonymous	No	**39**	**None**
	**136**	**T > C**	**18**	Synonymous	No	**39**	**None**
	313	A > G	2	Synonymous	No	4	A1
	326	A > G	4	Synonymous	No	9	A1
	**341**	**T > C**	**20**	Synonymous	No	**43**	**None and A1**
	370	G > A	1	Synonymous	No	2	A1
	397	A > G	2	Synonymous	No	4	A1
E7	628	T > C	2	Synonymous	No	4	A2
	**675**	**T > C**	**8**	Synonymous	No	**17**	**A1 and A2**

Note: % represents the proportion of the sequences where the E6 and E7 SNPs were found from the HPV35 study sequences (*N* = 46). Rows highlighted in bold indicate SNPs found in more than 10% of the sequences

## Discussion

Papillomaviruses have been identified in a wide array of vertebrates. With over 240 distinct types classified in 37 genera, papillomaviruses are one of the most successful families of vertebrate viruses [30]. They have been, and continue to be, an astonishing evolutionary success. HPV 33 and 35 are among the least phylogenetically studied despite their classification as high-risk types and group 1 carcinogen status [30, 31]. Both genotypes belong to the family of Alpha-papillomaviridae, specifically the Alpha 9 species. The International Agency for Research on Cancer (IARC) concluded that these genotypes have consistent and sufficient epidemiological, experimental and mechanistic evidence of carcinogenicity in humans for cervical cancer, as well as the HPV 31, HPV 39 and HPV 45 [31, 32]. Other carcinogenic HPV types are also implicated in cervical cancer, although they are less frequently detected compared to HPV 16, 18, 31, 39, and 45 [32].

In this study, 62% of HPV 33-positive participants were women with HSIL, confirming previous reports linking HPV 33 to severe cervical abnormalities. In sub-Saharan Africa, there is a lack of studies conducted to characterise the HPV 33 relationship with cervical abnormalities. Some studies conducted in Asia showed that in some cases, HPV 33 is more prevalent than HPV 18 in women with cervical lesions, being the second most common after HPV 16 [33, 34]. In the study conducted by Wang et al. (2010) on 1387 women diagnosed with cervical intraepithelial neoplasias (CIN 2 +), where hr-HPV positivity was found to be 91.6%, the types most commonly associated with CIN 2 + was listed as HPV 16, 58, 33 with 59.3%, 14.4%, and 10.0%, respectively [35]. In a study conducted by Purut and Uçkan (2023), on women with cervical intraepithelial lesions, the colposcopy results showed HPV 16 positivity in 43.3%, HPV 33 positivity in 30% and HPV 18 positivity in 10% of the patients with CIN2 + and above lesions [33].

Additionally, HPV 33 positivity has been linked to a higher incidence of CIN2 + lesions compared to lower-grade abnormalities [41]. Some studies indicate that HPV 33 is an independent predictor of CIN2 + lesions, with a fivefold increase in CIN2 + risk among HPV 33-positive women [41, 42]. This supports the hypothesis that HPV 33 plays a significant role in cervical malignancy development [42, 43]. In this study, HPV 33 European variants (A1 and A2 sublineages) were the most prevalent (38%), with the A1 sublineage accounting for 31%. The African 2 variant (B1 sublineage) was found in 19% of cases. Previous studies have identified A and B as the two predominant HPV 33 lineages [7, 11]. A study by Chen et al. (2013), which analysed HPV 33-positive cervical samples from the International Agency for Research on Cancer (IARC) biobank, found that A1, A2 and B sublineages were the most prevalent [11]. The A1 sublineage was strongly over-represented in cervical cases compared to controls in both Africa and Europe [11]. In 2011, Chen et al. sequenced the complete 8 kb genomes of 120 alpha-9 types (HPV 31, 33, 35, 52, 58 and 67) in samples collected from women from different African, Asian and American countries, to capture maximum viral heterogeneity and variations [10]. The results obtained for HPV 33 positive cases were the same as our present study results, where most of the study subjects clustered into the A1 sublineage.

In this study, the HPV 33 E6 region had twice as many SNPs as observed in the E7 region, and those SNPs were mostly found in the 11 samples that did not cluster with any of the reference sequences under investigation. Previous studies suggested that the HPV 33 E7 region is more conserved than E6 and, as a result, is considered a more suitable target for the diagnostic detection of HPV 33 [7, 19]. The intratypic variations observed in HPV 33 E6 and E7 can provide helpful information for identifying and distinguishing the known and the new HPV genotypes.

HPV 35 is frequently identified in women with cervical dysplasia and cervical cancer across multiple regions, including America, Asia and sub-Saharan Africa [12, 13, 36]. Consistent with previous research, our results confirm that HPV 35 exhibits lower genetic variation compared to other hrHPV types [31]. Consistent with the low level of genetic variation, HPV 35 is only divided into two sublineages, A1 and A2, rather than separate variant lineages, which are defined as requiring greater than 1.0% difference between complete genomes [37].

In this study, 78% of HPV 35 isolates clustered within the A1 sublineage, with 38% of these found in women with HSIL. Several studies have evaluated the association of HPV 35 A1 sublineage with the risk of cervical abnormalities. For example, a 7-year longitudinal study in Costa Rica found that the HPV 35 A1 sublineage may be associated with a higher risk of persistence and the development of CIN3 + compared to the A2 sublineage [35].

However, a study in the U.S. following up women every six months for two years identified no difference in cervical lesion risk between HPV35 sublineages [38]. In a Zimbabwean HPV 35-positive cohort, two of the five whole-genome sequences associated with HSIL clustered within the A2 sublineage [38]. Thus, it was suggested that future large-scale studies including HPV whole genome sequencing would be required to determine whether differential cervical cancer risk is associated with HPV 35 sublineages, and/or individual viral variants, including single-nucleotide changes and insertions/deletions [38, 39, 40].

It is suggested that the relationship between the European A2 lineages with the women of African ancestry could be a consequence of thousands of years of virus-host interactions, where certain lineages of HPV 35 became better adapted and able to persist in these women. Additionally, a likely explanation is that a genetic bottleneck due to the different out-of-Africa events and/or gene introgression events from archaic human populations may have rendered modern humans of non-African ancestry less susceptible to infection and/or progression with HPV35, and specifically the A2 sublineage [31].

Contrary to the high genetic variation observed between variants of other high-risk HPV types, the low phylogenetic distance between HPV 35 variants presented in this study is consistent with what is commonly observed in other populations around the world, with most HPV 35 variants clustering between only two sublineages (A1 or A2 sublineage) [10, 32]. Furthermore, in our study subjects were observed a significant diversity in the genealogical distribution since they are clustered among the A1 sublineages of the HPV 35 reference sequences isolated in countries such as Zambia, South Africa, Costa Rica, Togo and Zimbabwe.

HPV 35 is one of the most genetically conserved hrHPV types since its genome is relatively stable compared to other hr-HPV genotypes [10, 39]. In this study, three E6 SNPs (127 T > C, 136 T > C, and 341 T > C) were the most prevalent (39%) and were previously identified in other studies conducted in populations from the Americas, Asia, and Africa [31, 38, 40, 41]. In the E7 region, only one SNP (675 T > C) was found commonly. However, none of these SNPs were previously described or known to affect oncogenicity or immune escape.

This study found significant genetic variation in the HPV 35 E6 oncogene compared to the E7, a finding previously observed in several studies. For example, Mboumba Bouassa et al. (2024) explored the genetic variability of clinical strains of HPV 35 isolated from HIV-negative heterosexual adult women living in N’Djamena, Chad and HIV-infected men having sex with men (MSM) in the Central African Republic, where HPV 35 is highly prevalent. Their findings demonstrated that HPV 35 exhibited a higher rate of genetic variability in the E6 oncogene, while E7 sequences were highly conserved [40].

In this study, the most prevalent SNPs in the E6 gene were primarily found in isolates that did not cluster with any of the investigated reference sequences. Most of these SNPs were previously detected in A2 HPV 35 sublineages in whole-genome sequencing studies [38, 39, 40]. Evolutionary divergence in HPV variant lineages is common and is associated with different cancer risks, ethnicity, and geographical distribution [10, 30]. However, it is well established that the HPV 33 and 35 genotypes share a common ancestor with HPV 16, making them related within the same genotypes’ clade [10].

HPV 16 and HPV 18 sublineages have also been associated with cervical precancer/cancer in certain ethnic groups [42], and a viral-host interaction has been posited for HPV 16, where both modern-human migration and reproductive events, with introgression of immune-related alleles, confer a host niche adaptation, potentially influencing phenotype, such as cervical cancer [42, 43]. To add to this, Phylogenetic analysis in African HPV sequences demonstrated that the African HPVs share genetic ancestry with European sequences, whereas American isolates are less closely related. Migration analysis revealed a significant asymmetry in HPV flow, with migration rates from Africa to Europe [44].

Some study isolates did not cluster with any of the reference sequences under investigation, highlighting the need for whole-genome sequencing to improve variant characterisation [45]. Our findings were strictly based on a portion of the HPV genome. This fact does not allow us to classify the non-clustering subject as a novel HPV sublineage, recombinants, as well as to define whether the SNPs could be an immune escape, or they can interfere and impact the current HPV vaccines. However, due to the importance of E6 and E7 as oncogenes, future studies are needed to clarify the potential function of these variants. Additionally, our relatively small sample size limits statistical power, and future studies with larger cohorts could provide stronger associations between HPV 33 and HPV 35 sublineages and cervical cancer risk. This is a cross-sectional study, and this fact could be a limitation for the causal inference between variants and lesion severity in the studied subjects. Furthermore, our study subjects were collected only in two countries (South Africa and Mozambique), a fact that represents a geographical scope limitation, hence reducing our findings.

The low genetic diversity of HPV 35 isolates aligns with previous studies. However, given that some isolates in the present study did not cluster with the reference sequences, we speculate that whole-genome sequencing analysis would give a comprehensive understanding of this genetic diversity.

This study confirms a high prevalence of European HPV 33 and 35 variants. Taking into consideration that HPV 35 is still not targeted by the current HPV vaccines, further research is required to consider whether the addition of HPV 35 to the next generation of vaccines would result in a meaningfully broader coverage for African women. Vaccination and screening approaches that cover all the highest-risk types are needed for optimal global prevention coverage. Taking into consideration the present study’s genetic diversity evidence associated with a high burden of cervical cancer in Africa, prevention programs in populations without quality screening, the addition of HPV 35 to HPV vaccines would result in improved cervical cancer prevention. The current study findings add to the information on the variation in E6 and E7 of two important HPV types known to play an essential role in a significant number of African cervical cancers. This will help to inform the design of therapeutic vaccines for this region.

## Electronic supplementary material

Below is the link to the electronic supplementary material.

Supplementary Material 1: HPV 33 SNPs distribution according to the cytology

## Data Availability

Data is provided within the manuscript or supplementary information files.

## Abbreviations

CC: Cervical cancer
CIN: Cervical intatraephitelial neoplasia
E1-7: HPV early regions
DNA: Deoxyribonucleic acid
HSILs: High–grade squamous intraepithelial lesions
HPV: Human papillomavirus
hr-HPV: High–risk Human Papillomavirus genotypes
IARC: International Agency for Research on Cancer
KSD: King Sabata Dalindyelo health facility
L 1-2: HPV late region
MSM: Men having sex with men
MZ: Mozambican health centres
NCBI: National Centre for Biotechnology Information
NILM: Negative for intraepithelial lesion or malignancy
NM: Nelson Mandela Health Centre
ORFs: Open reading frames
PCR: Polymerase chain reaction
QC: Quality control
SAV: Sequencing analysis viewer
SNPs: Single–nucleotide polymorphisms
